# Perlite is a suitable model material for experiments investigating breathing in high density snow

**DOI:** 10.1038/s41598-022-06015-y

**Published:** 2022-02-08

**Authors:** Karel Roubik, Karel Sykora, Ladislav Sieger, Vaclav Ort, Lenka Horakova, Simon Walzel

**Affiliations:** 1 Department of Biomedical Technology, Faculty of Biomedical Engineering, Czech Technical University in Prague, Prague, Czech Republic; 2 Military Department, Faculty of Physical Education and Sport, Charles University, Prague, Czech Republic; 3 Department of Adapted Physical Education and Sport Medicine, Faculty of Physical Education and Sport, Charles University, Prague, Czech Republic; 4 Department of Physics, Faculty of Electrical Engineering, Czech Technical University in Prague, Prague, Czech Republic

**Keywords:** Physiology, Medical research

## Abstract

Outdoor breathing trials with simulated avalanche snow are fundamental for the research of the gas exchange under avalanche snow, which supports the development of the international resuscitation guidelines. However, these studies have to face numerous problems, including unstable weather and variable snow properties. This pilot study examines a mineral material perlite as a potential snow model for studies of ventilation and gas exchange parameters. Thirteen male subjects underwent three breathing phases—into snow, wet perlite and dry perlite. The resulting trends of gas exchange parameters in all tested materials were similar and when there was a significant difference observed, the trends in the parameters for high density snow used in the study lay in between the trends in dry and wet perlite. These findings, together with its stability and accessibility year-round, make perlite a potential avalanche snow model material. Perlite seems suitable especially for simulation and preparation of breathing trials assessing gas exchange under avalanche snow, and potentially for testing of new avalanche safety equipment before their validation in real snow.

The study was registered in ClinicalTrials.gov on January 22, 2018; the registration number is NCT03413878.

## Introduction

A number of outdoor breathing trials with healthy volunteers has been conducted in order to investigate factors affecting the survival of victims covered with avalanche snow^1–7^. The main research has been focused on the gas exchange occurring in the air contained within the snow^1,3–6^ and the role of an ‘air pocket’ in gas exchange^1–3,7^. This research is fundamental for development of the international resuscitation guidelines^8,9^.

Usually, breathing experiments with volunteers are organized in the mountain terrain with simulated avalanche snow. Even in this environment it is complicated to include all factors affecting the survival (e.g. mechanical factors, hypothermia, etc.). The design and preparation of these experiments are very demanding also due to a limited accessibility of snow and due to unpredictable weather conditions.

It is a well-accepted presumption that the physical properties of snow can contribute to the rate of avalanche victim survival^1,4,10^. However, the snow properties differ among the published studies. The average avalanche snow density ranges from 200 kg m^−3^ (dry snow) to 550 kg m^−3^ (wet or dry)^11^. In their studies, Brugger et al*.* used snow with median density of 376 kg m^−3^ (range 144–546 kg m^−3^)^1^, Roubik et al*.* used snow of a similar density with much smaller variance (380 ± 14 kg m^−3^)^3^. Strapazzon et al*.*^4^ even conducted three series of experiments, time separated, with the median snow density of 364 kg m^−3^ (range 155–481 kg m^−3^).

The field environment poses additional challenges to the experiments. For example, standard medical devices used outdoors can be a source of erroneous readings of the physiological parameters^12^. Some of the limitations can be overcome in a special laboratory capable of simulating various aspects of the mountain environment^13^.

Another approach could be finding a stable material that can simulate avalanche snow in a laboratory environment. This snow modeling material could facilitate research focused on an isolated factor of gas exchange of avalanche buried victims and aid testing of protocols for breathing experiments before their implementation in real snow.

A small prospective laboratory study was conducted by Roubik et al*.*^14^ in order to select a material which could serve as a possible model of avalanche snow for studies of ventilatory and gas exchange parameters. From three loose porous materials (perlite, wood shavings and polystyrene), perlite was found as the most suitable one to simulate avalanche snow because the resulting concentrations of breathing gasses copied concentrations measured during breathing into real snow. Furthermore, perlite has favorable properties, mainly the homogeneity and it is easy to manipulate with. By changing the water content in perlite, they were able to modify its physical properties and thus affect the gas exchange when simulating a burial in this material.

To our best knowledge, there has not been published other study dealing with breathing under different materials simulating the avalanche snow. There have been only reported cases of victims covered with rubble, mainly following an earthquake^15^, or buried in sand^16^.

The aim of the present study was to find a suitable material as a surrogate of avalanche snow for breathing experiments aimed at studying ventilation physiology and factors affecting survival of victims covered with avalanche snow.

## Methods

The study protocol was approved by the Institutional Ethical and Review Board of the Faculty of Biomedical Engineering, Czech Technical University in Prague (No. A001/018, issued on January 22, 2018), and written informed consent was obtained from the volunteers before enrollment in the study. All methods were performed in accordance with the relevant guidelines and regulations. The study was registered in the ClinicalTrials.gov register (NCT03413878) on January 22, 2018, before enrollment of the first volunteer.

The prospective interventional randomized double-blind crossover pilot study was conducted in Spindleruv Mlyn, Krkonose Mountains, Czech Republic, at the altitude of 762 m above sea level on January 29, 2018 – February 1, 2018.

### Subjects

Thirteen male volunteers participated in the study, eleven were included in the final analysis. The number of participants was selected as a standard number of subjects included in published studies with a similar scope and design^1–4,6,7,17–19^. All eligible volunteers were members of the Czech Armed Forces and students at the Military Department of the Faculty of Physical Education and Sport, Charles University in Prague.

All study subjects were fit and well, classified according to the American Society of Anesthesiologists as ASA I^20^. They underwent an initial examination performed by an experienced physician, including assessment of the past medical history, smoking history, physical examination and spirometry (MSP1, Mesit, Uherske Hradiste, Czech Republic). The exclusion criteria were the Tiffeneau Index less than 0.70, any acute respiratory infection, and a history of cardiovascular or respiratory disease.

### Study design

Each volunteer underwent three phases of the experiment: phase “S”—breathing into snow, phase “PD”—breathing into dry perlite, and phase “PW”—breathing into wet perlite (CONSORT flowchart in Supplementary Fig. 1). The volunteers were randomly divided into six equal groups in two steps. In the first step, the volunteers were divided into three groups, each starting the experiment with one of the three tested materials. In the second step, the volunteers were randomly assigned an order of the two remaining tested materials. As a result, every subject underwent all three experimental phases (S, PD and PW), but in randomized orders, i.e., 39 individual breathing experiments were performed.

The allocation was made using computerized random numbers by an assistant not taking part in further research. The volunteers and the investigators directly conducting the experiment did not know whether the prepared experiment involved S, PD or PW breathing. At least an 8-h recovery interval was included in between breathing phases of each subject.

### Equipment

**Figure 1:**
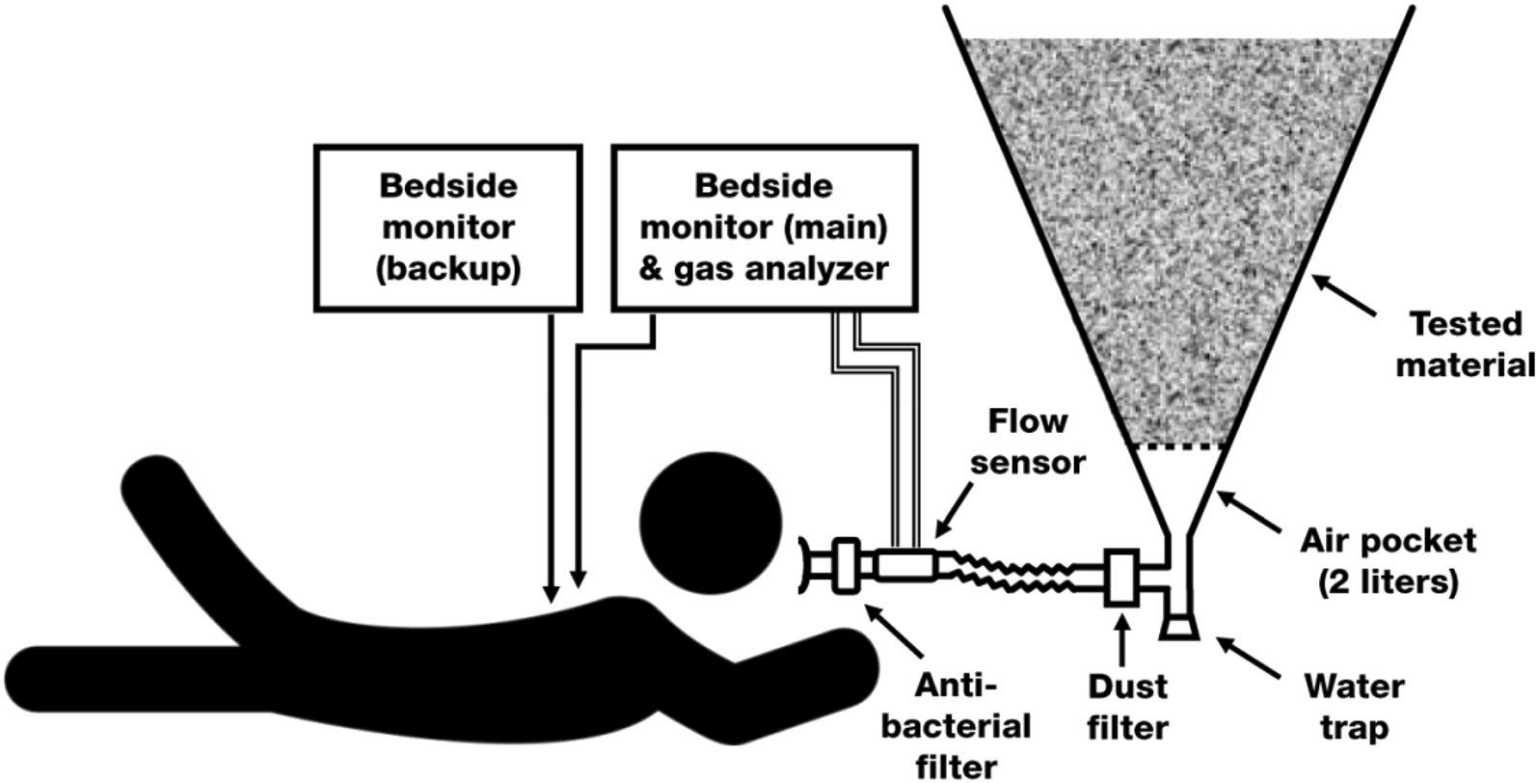
Experimental setup (modified from^14^, with permission).

Continuous monitoring of all study subjects was performed throughout the whole experiment. Before initiation of each breathing experiment, the subject was attached to vital sign monitors. Datex-Ohmeda S/5 (Datex-Ohmeda, Madison, WI, USA) served as a primary monitor, which monitored and recorded the following physiological parameters: electrocardiography (ECG), heart rate (*HR*), non-invasive blood pressure (*NIBP*–intermittently in two-minute intervals), and peripheral blood oxygen saturation (*SpO*_*2*_). Using a respiratory sensor D-Lite (Datex-Ohmeda, Madison, WI, USA), connected between the mouthpiece and the tubing leading to the cone (Fig. 1), ventilation parameters were obtained, namely: breathing frequency (*BF*), tidal volume (*V*_*T*_), airway pressure (*Paw*) and airflow (*Qaw*) measured at the airway opening. The spirometry sensor D-Lite also provided a gas sampling port for the Datex-Ohmeda S/5 monitor measuring inspiratory and end-tidal fractions of oxygen (*FiO*_*2*_*, EtO*_*2*_), carbon dioxide (*FiCO*_*2*_*, EtCO*_*2*_), and nitrous oxide (*FiN*_*2*_*O*, *EtN*_*2*_*O*), using an E-CAiOVX (Datex-Ohmeda, Madison, WI, USA) anesthesia and spirometry module. The analyzer was calibrated before each experimental day using a standard calibration gas. Apart from the monitor Datex-Ohmeda S/5, there was another vital sign monitor, CareScape B650 (GE Healthcare, Helsinki, Finland), used as a backup *SpO*_*2*_ and *HR* monitor of the subjects.

The vital sign monitors were connected to laptop computers for data collection (S/5 Collect software, Madison, WI, USA). All the devices were placed in a tent equipped with an electric heater and the gas sampling lines were wrapped in a polyurethane foil for insulation and supplemented with a heated wire in order to prevent condensation and freezing of water in the tubing.

The experiments were recorded on three independent camcorders; the first one continuously recorded the vital sign monitor screens, the second one recorded the study subject and the third one the overall situation on the site. The video and audio recording was intended as a backup of the data recorded by the laptop computer and written protocols. This enabled reviewing the situation on the site later during the data processing and evaluation. The investigators communicated via PMR (private mobile radio) strictly adhering to the protocol protecting the double-blind design of the study.

### Protocol

The subject was connected to vital sign monitors and laid down in a prone position on an insulated mat in front of a custom-made apparatus described below. The prone position was chosen as it is the most common pose of buried avalanche victims^21^.

At the initiation of each breathing phase, the subject had a nose clip properly placed and was attached to a mouthpiece so that he was able to breathe only through the specially designed breathing circuit. The mouthpiece was at this point connected only to the flow sensor, not to the cone yet, allowing measurement of respiratory gasses and baseline ventilation parameters. After reaching stable ventilation parameters, especially a steady tidal volume and breathing frequency (after approx. 5 min), the subject was connected to the cone and the breathing experiment was commenced. At the same time, nitrous oxide administration to the vicinity of the subject airways and the breathing circuit was initiated for potential leak detection and all the physiological and study parameters continued to be recorded and observed.

Safety of the volunteers during the experiment was assured by advanced simultaneous monitoring and continuous evaluation of their physiological parameters by the attending physician with a specialty training in anesthesia and emergency medicine. Throughout the whole experiment, a clinical assessment of consciousness level of the volunteer was performed: the physician asked the subject to calculate simple mathematical operations and to show the result using his fingers which were not attached to the pulse oximeter probes. The physician could terminate the experiment at any time.

The criteria for termination of the breathing experiment were: a request of the subject, a supervising physician's command, reaching of the safety limit (end tidal carbon dioxide *EtCO*_*2*_ of 62.5 mmHg), an accidental disconnection or a detection of the tracing gas (N_2_O) in the breathing circuit. The participant was disconnected from the test material and allowed to breathe ambient air through the mouthpiece with the respiratory sensor still attached and while the ventilatory and physiological parameters were still recorded. After further 2 min, when all parameters stabilized and returned close to the baseline, the subject was disconnected from the mouthpiece and the test phase was ceased.

### Preparation of the breathing apparatus

The apparatus, depicted in Fig. 1, consisted of a stiff metal inverse cone with a total volume of 65 L, 1 m high, the apex angle was 28 degrees. The inner wall of the cone was lined with a 1 mm thick polypropylene foil, which prevented freezing and heat conduction. The shape of the inverted cone as a snow container was chosen to prevent possible gas leakage along the walls of the container. If an air layer had begun to form near the walls of the container as the snow melted, it would had been eliminated immediately by the descent of the snow body deeper into the cone due to gravity. The bottom part of the cone was equipped with a two-liter air pocket and the rest of the cone was filled with 54 L of the tested material (more details of the cone construction are described in^14^). The cone tip was connected to the rest of the breathing apparatus, which consisted of a dust filter, a 12 cm corrugated tube, a flow sensor with a gas sampling port, an antibacterial filter and a mouthpiece. There was also a water trap for draining off any excessive water.

All parts of the breathing circuit were designed in an attempt to minimize the equipment dead space and resistance to breathing effort. The dead space of the whole breathing circuit was 220 mL and its airflow resistance was 475 Pa⋅s/L measured at the flow rate of 60 L/min according to EN ISO 8835–2. Owing to the fact that some components of standard anesthetic tubing were modified for this experiment, it posed a risk of leaks of the gas from the system. As it is a standard practice to check for leaks of breathing systems in anesthesiology and intensive care, also in this experiment a system for leak detection was used, utilizing nitrous oxide (N_2_O) as a ‘tracing gas’. This technique has already been used during a similar study^3^. This easily detectable gas was administered via a 6 mm flexible tube into the vicinity of participant’s airways, breathing mouthpiece and along the breathing circuit at flow 20 L·min^−1^. The gas analyzer of the anesthetic monitor Datex-Ohmeda S/5 was sampling the gas from inside of the breathing circuit and thus was able to detect even a minimal concentration of nitrous oxide. The presence of the nitrous oxide in the system suggested either a leak in the tubing system, or a situation when the test subject inhaled gas not directly from the mouthpiece, but around the mouthpiece or through the nose, which was not properly sealed by the nose clip. The positive detection of N_2_O in the system was a reason to immediately stop the particular breathing phase.

### Tested materials

As a material which could serve as a model of the snow, perlite was chosen, based on a pilot study with several other materials^14^. Perlite is an amorphous industrial mineral and also a commercial product, useful for its low density and ability to bear a relatively high amount of water. The grain size of the perlite specified by the manufacturer was 1–3 mm (“Expandovany perlit EP AGRO”; Perlit ltd., Senov u N. Jicina, Czech Republic). It is a non-toxic material, perlite dust is in most countries listed as a “nuisance dust”^22^. However, as a special precaution, an extra High Efficiency Particulate Air (HEPA) filter (Servo Duo Guard, MAQUET, Solna, Sweden) was inserted between the cone filled with the tested material and the rest of the breathing apparatus (as showed in Fig. 1).

The material was used in two forms—dry and moisturized. The moisturized perlite was a mixture of dry perlite and water in the proportion of 100:20 by volume. The mixture was then left to settle and mixed several times a day to ensure a uniform composition.

Filling of the cone with snow was performed gradually by adding and compacting small portions of homogeneous snow collected from a depth of 20–50 cm from the surface. The snow was identical throughout the depths. Both dry and wet perlite are non-clogging materials; thanks to this, they could be poured into the cone at once. The density of all materials was measured by weighing the whole cone filled with the tested material (54 L) before every experimental phase. The porosity of the snow was then derived from the knowledge of the ice density^23^. The porosity of perlite was quantified by filling a calibrated cylinder (1.6 L) full with the tested material and topping up with water and then the whole cylinder was weighted on the scales. Porosity was then calculated as:

$$porosity=1- \frac{{m}_{mat}-{m}_{0}}{{m}_{tot}- {m}_{0}},$$

where ${m}_{mat}$ is weight of the cylinder filled with the tested material, ${m}_{0}$ is weight of the empty cylinder and ${m}_{tot}$ is weight of the cylinder filled with the tested material and then flooded fully by water. The density and porosity of the wet and dry perlite was experimentally measured before every breathing experiment of the volunteers.

Throughout the whole study, the snow temperature was between − 0.8 and 0 °C in the snow profile from the surface to the depth of 20 cm (not used for the experiments), and the temperature of snow was stable at 0 °C between 20 and 50 cm below the surface (this snow was used for the experiments). The snow used during the experiment was classified according to the International classification for seasonal snow on the ground^24^ as wet snow without any impurities. Snow density was 542 ± 32 kg m^−3^, grain size was ‘very coarse’ (2–3 mm), snow hardness corresponded to ‘soft snow’ (4F), grain shape was classified as ‘melted forms’ (clustered rounded grains). The average ambient temperature during the breathing experiments varied from 0 to 3 °C. The minimum temperature during nights was − 5.4 °C. Atmospheric pressure was 91.8 ± 1.2 kPa (range from 90.5 to 93.0 kPa).

### Data processing and statistics

The data from all 13 volunteers and tested materials were included in the analysis of the time to phase termination presented by the Kaplan–Meier plot. The Mantel–Haenszel test was used to statistically compare the cumulative termination rates between the individual phases (S, PD and PW). From the whole group of 13 volunteers, only 11 (*N* = 11) volunteers fulfilled the following criteria and were included in the final data analysis: sustained breathing during all three phases for at least 240 s and high-quality data recorded during all phases. Therefore, the number of participants included in the final statistical analysis was 11.

The statistical difference between the physiological parameters (*SpO*_*2*_*, FiO*_*2*_*, EtO*_*2*_*, FiCO*_*2*_*,* and *EtCO*_*2*_) during S, PD and PW phases were calculated in 15 s intervals from the beginning of the experiment (i.e., connection of the breathing circuit to the cone with the tested material). The values were expressed as mean ± standard deviation and presented in graphs. The statistical significance of the difference was tested using the two-way ANOVA for repeated measures with Tukey HSD post-hoc tests (STATISTICA 7.1, StatSoft, Tulsa, OK, USA) after the Shapiro–Wilk data normality test. *P* < 0.05 was considered as statistically significant.

## Results

The tested materials were characterized before every breathing experiment. Their basic properties are presented in Table 1.

**Table 1:** Basic properties of the tested materials.

Material	Symbol	Density (kg m^−3^)	Porosity (–)
Snow	S	542 ± 32 (510–574)	0.41 ± 0.04 (0.37–0.44) ^a^
Dry perlite	PD	130 ± 1 (128–132)	0.64 ± 0.01 (0.63–0.65)
Wet perlite	PW	333 ± 3 (328–338)	0.50 ± 0.02 (0.48–0.52)

The values are presented as mean ± standard deviation and range (minimum–maximum).

^a^The porosity of the snow was calculated from its density^23^.

**Figure 2:**
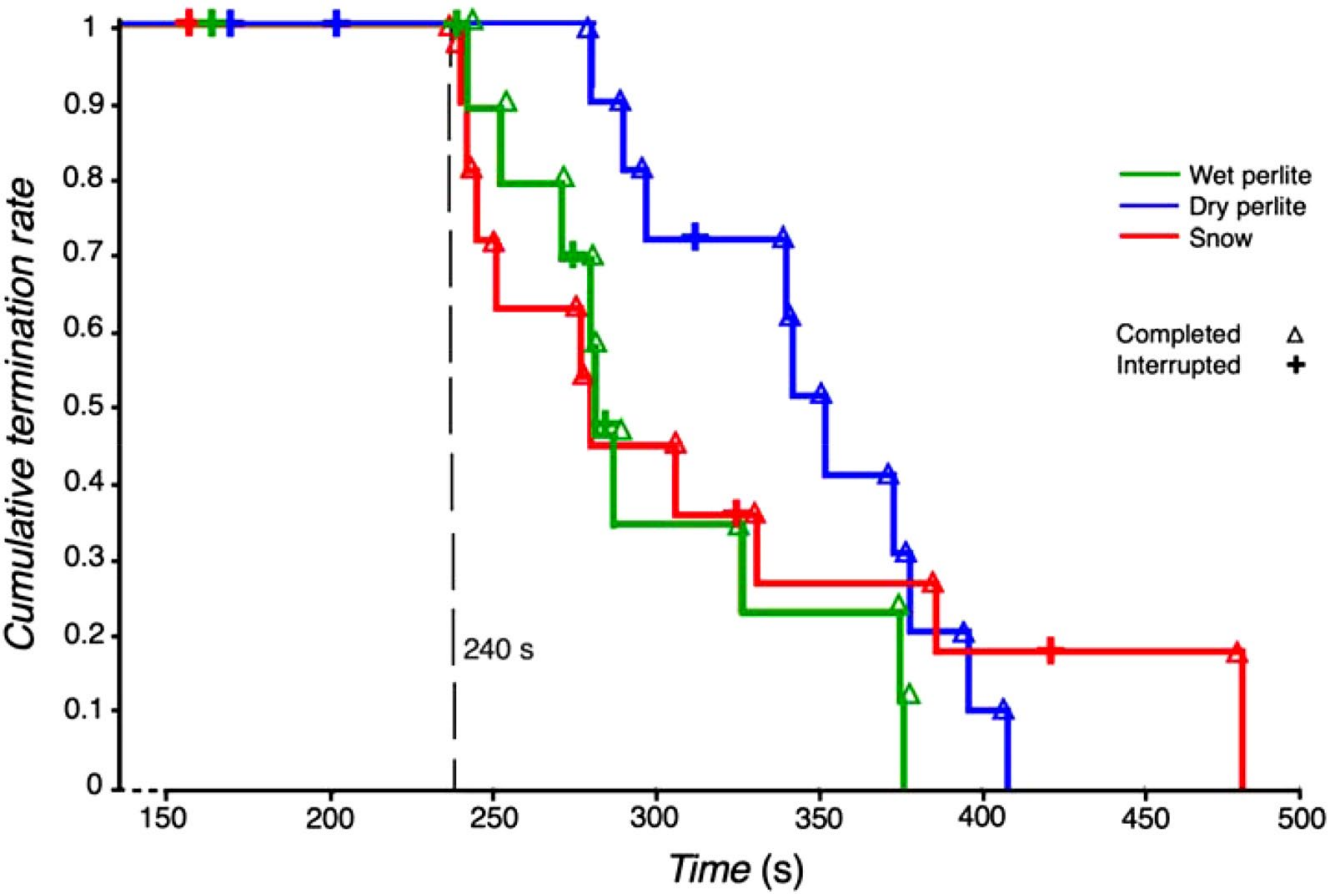
Time to breathing experiment termination for the three different phases: snow (S), dry perlite (PD) and wet perlite (PW). The term “completed” means that the subject terminated the experiment upon his own request, or the experiment was terminated by the supervising physician based on the clinical assessment of the subject. The term “interrupted” means that the experiment was terminated due to accidental disconnection, or detection of N_2_O in the breathing circuit.

The Kaplan–Meier plot of time to the breathing experiment termination for the whole group of 13 volunteers and for all the three phases is presented in Fig. 2. The characteristics of the full group of volunteers analyzed using the Kaplan–Meier plot are presented in the first data column in Table 2. None of the experiments lasted longer than 418 s, except one in snow (480 s). At the same time, none of the breathing phases included in the study terminated earlier than at the 240th second. For this reason, further data processing was performed using the first 240 s of the breathing phases (i.e., 240 s since connection of the breathing circuit to the cone with the tested material). The reasons for termination of the breathing phases are listed in Table 3. The cumulative termination rate of participants breathing into snow and wet perlite was similar. As the time in the breathing phase increased, the later termination of the subjects was more frequent when breathing into snow or dry perlite. Based on the Mantel–Haenszel test, there is no statistically significant difference (p-value 0.146) in the cumulative termination rates between the individual phases (S, PD and PW).

**Table 2:** The basic characteristics of the group of volunteers involved in the breathing experiment.

Parameter	All volunteers (*N* = 13)	Volunteers included in data analysis (*N* = 11)
*Age* (years)	22.8 ± 4.1 (20–35)	22.7 ± 4.7 (20–35)
*Weight* (kg)	80.8 ± 8.8 (66–103)	80.4 ± 9.9 (66–103)
*Height* (cm)	179.5 ± 5.0 (172–187)	179.4 ± 5.6 (172–187)
*BMI* (kg m^–2^)	25.1 ± 2.6 (22.3–33.3)	25.0 ± 2.9 (22.3–33.3)
*FEV1* (L)	4.6 ± 0.6 (3.3–5.4)	4.7 ± 0.7 (3.3–5.4)
*FVC* (L)	5.0 ± 0.8 (3.3–6.0)	5.0 ± 0.9 (3.3–6.0)
*FEV1*/*FVC*	0.93 ± 0.05 (0.84–0.96)	0.93 ± 0.05 (0.84–0.96)

The values are presented as mean ± standard deviation and range (minimum–maximum). *BMI* body mass index, *FEV1* forced expiratory volume in 1 s, *FVC* forced vital capacity.

**Table 3:** Reasons for termination of the breathing experiments and their frequency.

Termination of the breathing experiment	Category	Frequency
N_2_O in breathing circuit	Interrupted	5 (13%)
Accidental disconnection	Interrupted	5 (13%)
Supervising physician	Completed	5 (13%)
Subject's request	Completed	24 (61%)

Eleven (85%) of thirteen volunteers were included in the data analysis. The characteristics of the subgroup of 11 analyzed volunteers are presented in the second data column in Table 2. Two volunteers were excluded owing to the following reasons: one volunteer during the PD and PW phases prematurely terminated the breathing into the cone due to an accidental disconnection before the minimal period of 240 s. Another volunteer was excluded because of the presence of the tracing gas (N_2_O) in the breathing circuit in both S and PD phases and the breathing was also shorter than 240 s. There was no harm to any subject of the experiment.

**Figure 3:**
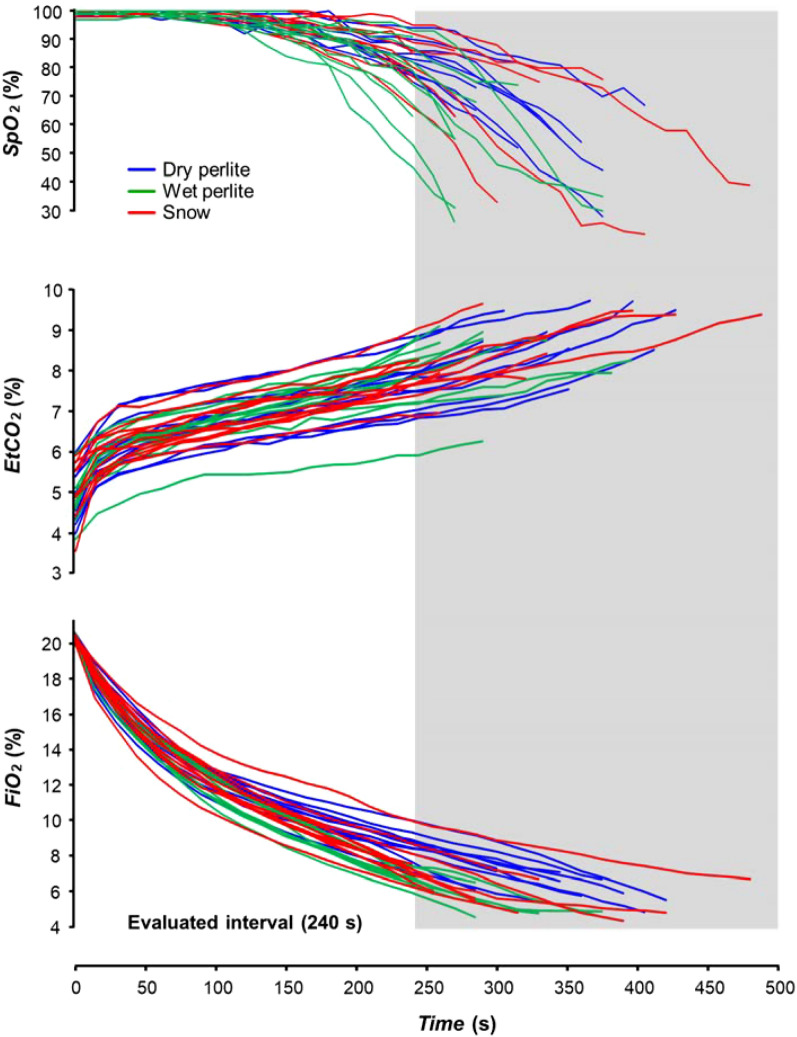
Individual curves of peripheral blood oxygen saturation (*SpO*_*2*_), end-tidal fractions of carbon dioxide (*EtCO*_*2*_) and inspiratory fractions of oxygen (*FiO*_*2*_) of each of the volunteers for all the three experimental phases: snow (S), dry perlite (PD) and wet perlite (PW).

The individual curves of the physiological parameters *SpO*_2_, * EtCO*_2_ and * FiO*_2_ of each of the volunteers involved in the study, color-coded for all three conditions (PD, PW and S), are presented in Fig. 3. The figure also indicates the interval of the first 240 s, which was used for the subsequent processing of the measured physiological parameters.

**Figure 4:**
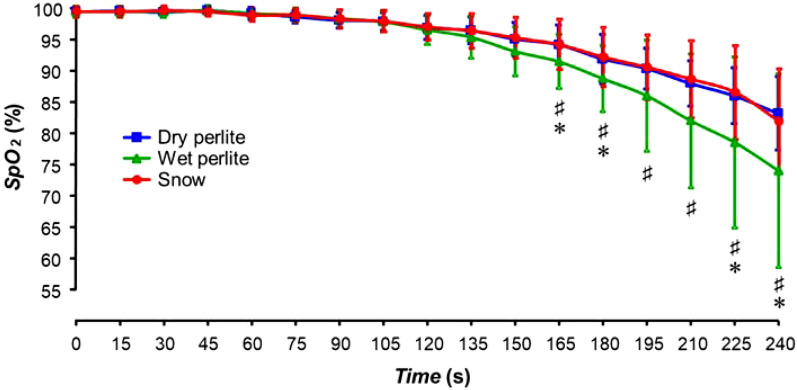
Peripheral blood oxygen saturation (*SpO*_*2*_) of the subjects during the three different phases: snow (S), dry perlite (PD) and wet perlite (PW). The symbol # represents statistically significant differences between snow and wet perlite, the symbol * represents statistically significant differences between dry perlite and wet perlite; *p* ≤ 0.05.

The statistically significant difference in *SpO*_*2*_ between the wet perlite and the dry perlite breathing phases appeared at 165 s and lasted until the end of the experiments, as depicted in Fig. 4. The desaturation defined as the decrease of the *SpO*_*2*_ curve for the breathing phase into dry perlite overlaps the course of the *SpO*_*2*_ curve for snow. Moisturizing of perlite had a significant effect on the rate of desaturation development.

**Figure 5:**
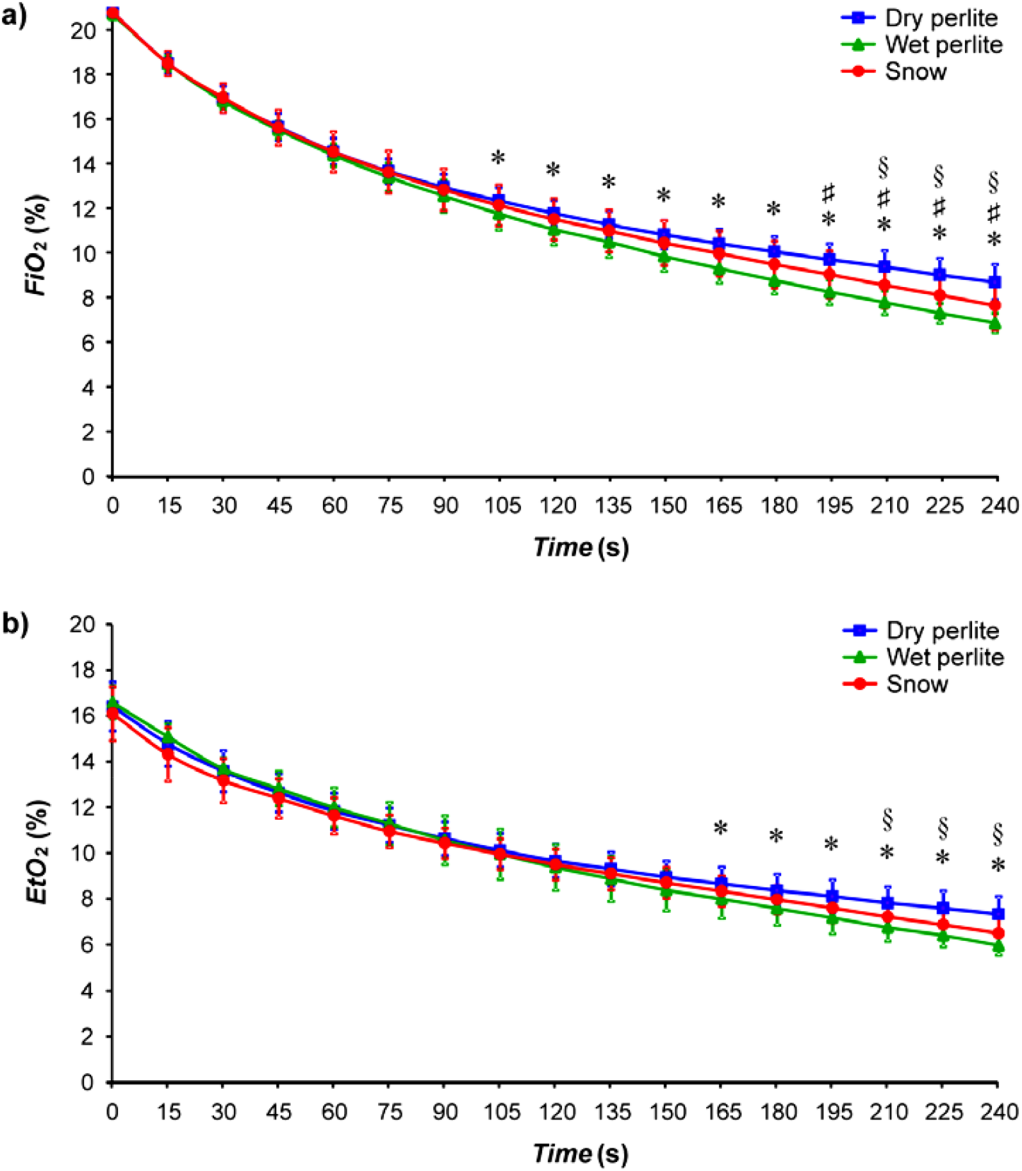
Inspiratory fractions of oxygen (*FiO*_*2*_) in (**a**) and end-tidal fractions of oxygen (*EtO*_*2*_) in (**b**) in the breathing gas during the three different phases: Snow (S), Dry perlite (PD) and Wet perlite (PW). The symbol * represents statistically significant differences between dry perlite and wet perlite, the symbol # represents statistically significant differences between snow and wet perlite, the symbol § represents statistically significant differences between snow and dry perlite; *p* ≤ 0.05.

Inspiratory and end-tidal fractions of oxygen (Fig. 5) were the highest for the dry perlite and the lowest for the wet perlite the whole time. The curve for snow is situated between these two mentioned materials for both inspiratory and end-tidal fractions of oxygen. Statistically significant differences in *FiO*_*2*_ were found between the dry perlite and the wet perlite breathing phases starting at 105 s, between the snow and the wet perlite at 195 s and between the snow and the dry perlite at 210 s. The statistically significant differences in *EtO*_*2*_ between the dry and the wet perlite occurred later, starting at 165 s.

**Figure 6:**
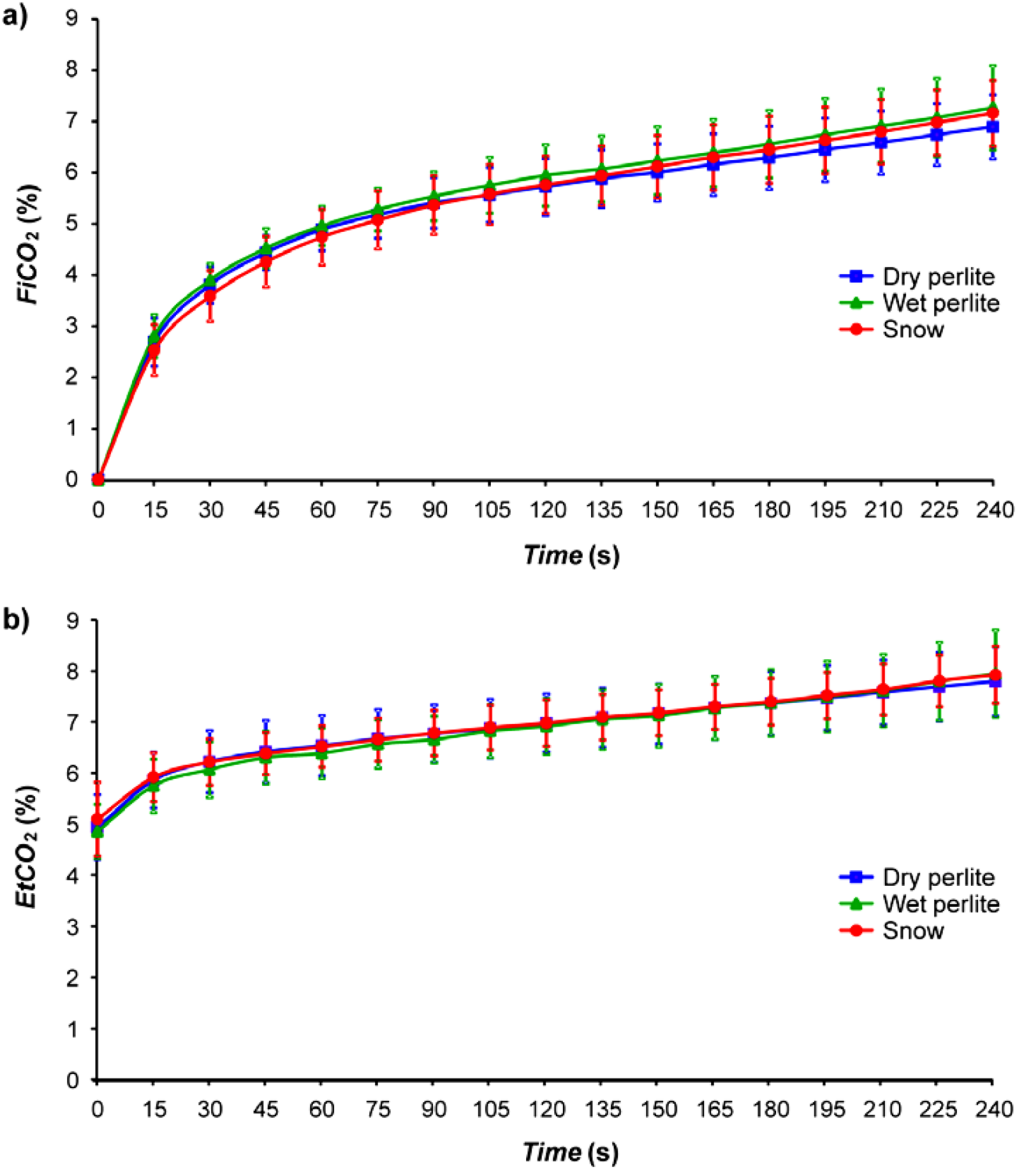
Inspiratory fractions of carbon dioxide (*FiCO*_*2*_) in a) and end-tidal fractions of carbon dioxide (*EtCO*_*2*_) in b) in the breathing gas during the three different phases: snow (S), dry perlite (PD) and wet perlite (PW). No statistically significant difference among the breathing phases was found.

For inspiratory and end-tidal fractions of carbon dioxide, there was not found any difference between any of the tested materials in the whole study. In the graphs (Fig. 6), all three curves overlap each other.

## Discussion

The main finding of this study is that the characteristics of perlite in terms of gas exchange are similar to high density snow used in this study and furthermore, the trends of gas exchange parameters in snow lie in between the wet and dry perlite trends.

Concerning the carbon dioxide, there were no differences in inspiration (*FiCO*_*2*_) and expiration (*EtCO*_*2*_) concentrations among tested materials. The trends of the curves representing *EtCO*_*2*_ during breathing into snow, wet and dry perlite overlapped each other and the same behavior was observed for *FiCO*_*2*_.

As for the oxygen, the parameters *FiO*_*2*_ and *EtO*_*2*_ differed for snow, wet and dry perlite, nevertheless the trends had a similar shape for all the materials and the curve representing snow lay in between the curves of wet and dry perlite.

The presented results support the idea of utilizing perlite as a suitable material that can substitute avalanche snow during laboratory experiments. The trends of the gas exchange parameters in all tested materials were similar or they slightly differed. When they differed, the curve for snow lay always in between the corresponding curves for wet and dry perlite.

According to the presented results we speculate that perlite can simulate the high density snow properties concerning the gas exchange. By a simple modification of the water content in perlite the gas exchange characteristics of this material can be adjusted.

The course of subject’s desaturation represented as an *SpO*_*2*_ drop was similar when breathing into dry perlite and into snow, and the addition of water to perlite caused a faster course of *SpO*_*2*_ desaturation. This finding further confirms that perlite is a suitable material simulating high density snow during breathing experiments and that the humidity of perlite slightly modifies its gas exchange properties.

The biggest advantage of perlite is its homogeneity and stability over time and thus also the reproducibility of the experiments. It is a very cheap material and easy to manipulate with. Even though perlite is reported as a non-toxic material^22^, a HEPA filter was used in the breathing apparatus in order to prevent inhalation of the dust particles by the subjects. The authors recommend using HEPA filters efficient in removing dust particles in the future experiments conducted with perlite.

Another advantage of perlite is the considerable capacity to hold water. The perlite water content can modify not only the properties of the material itself, but also its effect on respiratory gases (*FiO*_*2*_*, EtO*_*2*_) and peripheral oxygen saturation (*SpO*_*2*_) during breathing experiments.

These results are in concordance with the Kaplan–Meier plot demonstrating similar properties of perlite compared to high density snow; nonetheless, studying the maximum length of breathing to the experiment termination was not the aim of the study. Rather the aim was to attain a time segment of a sufficient length in order to study the concentrations of respiratory gasses and peripheral saturation *SpO*_*2*_ in all subjects.

The current study has several limitations. Firstly, the recruited volunteers were solely young male students from the Czech Armed Forces, well trained and highly motivated. However, it was our intention to select such a homogenous group of subjects in order to obtain concise results and also to have a smaller number of subjects because the experimental results then show smaller variance. Although male avalanche victims prevail over female in the studies^25–27^, there are further studies needed with different groups of subjects in order to generalize these results.

Secondly, factors affecting the survival of avalanche buried victims, such as mechanical factors, trauma, psychological stress, and hypothermia^28^, cannot be experimentally investigated in humans for ethical reasons^5^. Furthermore, the study was designed in order to eliminate a variety of negative cofactors listed above. As a consequence, the isolated effects of the gas exchange properties of different materials could be studied.

Another limitation is based on the published studies reporting survival time of real victims covered with avalanche snow. Approximately 10% of victims do not survive the first 15 min of the snow burial^29^ in Europe and 10 min^10^ in North America due to asphyxia. Nevertheless, the present study evaluates data from the first 4 min of each breathing experiment. Studying the difference in survival between the tested materials was not the aim of the study; rather, the study was designed to investigate differences in gas exchange between the tested materials. To be able to analyze possible differences of the tested materials in terms of gas exchange during breathing, it was necessary to have a sufficiently large group of subjects who sustained to breathe into the tested materials for the same time. The mentioned method of data processing, when only the initial segments of data records are statistically evaluated while the number of subjects remains constant, is common even in the case of breathing experiments in simulated avalanche snow, especially when the gas exchange parameters are studied as we did in our study. As an example, Brugger et al.^1^ statistically evaluated the development of SpO2 within 240 s from the beginning of the experiment, which is the same time period that we used in our study. To be able to make a proper and statically sound comparison, we tried to eliminate the known effects that can negatively affect the measurement and can increase the variance of data within the studied groups. Therefore, the experiment was properly prepared and conditions were continuously checked. For this reason, for example, we used a series of tests for tightness and gas leakage along the entire path of the breathing circuit (from its connection to a volunteer down to the wall of the cone) using N_2_O as a tracer gas. We believe that this care assured that the studied respiratory parameters are consistent within the studied groups (S, PD and PW) and show very small variances. This allowed to demonstrate the statistically significant differences in the properties of the studied materials (where they were present) even within a relatively short length of analyzed data.

Next, the study was conducted in stable weather conditions with certain physical snow properties (e.g., snow type and density, temperature, etc.). Moreover, only a single type of snow was available at the site of the experiment.

Finally, the study was conducted at a low altitude site with higher oxygen partial pressure compared to common avalanche accident locations. This might affect the absolute values of *FiO*_*2*_, *EtO*_*2*_ and *SpO*_*2*_, but the general trends of these parameters should not be affected significantly.

This study and the suggested avalanche snow model, i.e. perlite, is not intended for experiments modeling different snow properties. The snow properties themselves may have, according to recent research, an important effect on the survival time of avalanche victims^1,4,6,10^.

Another obvious limitation of perlite is that there is no interaction between breathing air and snow. In real cases, the snow transforms when it comes into contact with the warm air exhaled by the buried subjects. This property of perlite represents an advantage for the reproducibility of the experiments, but it also represents a limitation of the perlite as a surrogate material of snow.

The authors of the study see the possible application of this snow model material in preparation of breathing experiments, for testing of experimental protocols, technical equipment and specially designed breathing circuits preceding the field experiments in the mountain environment. It can also be used for studies on different population groups (i.e., with various physical condition, age, etc.) and for initial testing of new avalanche safety equipment. Last but not least, it can help to train the subjects in laboratory environment for outdoor experiments if this training is a part of the protocol.

## Conclusions

This is the first study to document that it is possible to use a material which could simulate some of the properties of avalanche snow in laboratory conditions. The study suggests that perlite can be used as a surrogate of high density snow for studying gas exchange. Although these laboratory experiments cannot fully replace field studies in the real environment, they may be very beneficial in preparation and methodology validation of the outdoor experiments. Furthermore, due to the stability and homogeneity of perlite, the experiments yield consistent and repeatable results.

## Supplementary Information

Supplementary Information.

## Data Availability

The datasets generated and analyzed during the current study are available in the repository at https://ventilation.fbmi.cvut.cz/data/.
