# Supplementary material for: Perlite is a suitable model material for experiments investigating breathing in high density snow
Source: Sci Rep. 2022 Feb 8;12:2070. doi: 10.1038/s41598-022-06015-y (PMC8827056; doi:10.1038/s41598-022-06015-y)
Supplement: Supplementary file 1 — Supplementary Information. [file 41598_2022_6015_MOESM1_ESM.pdf]

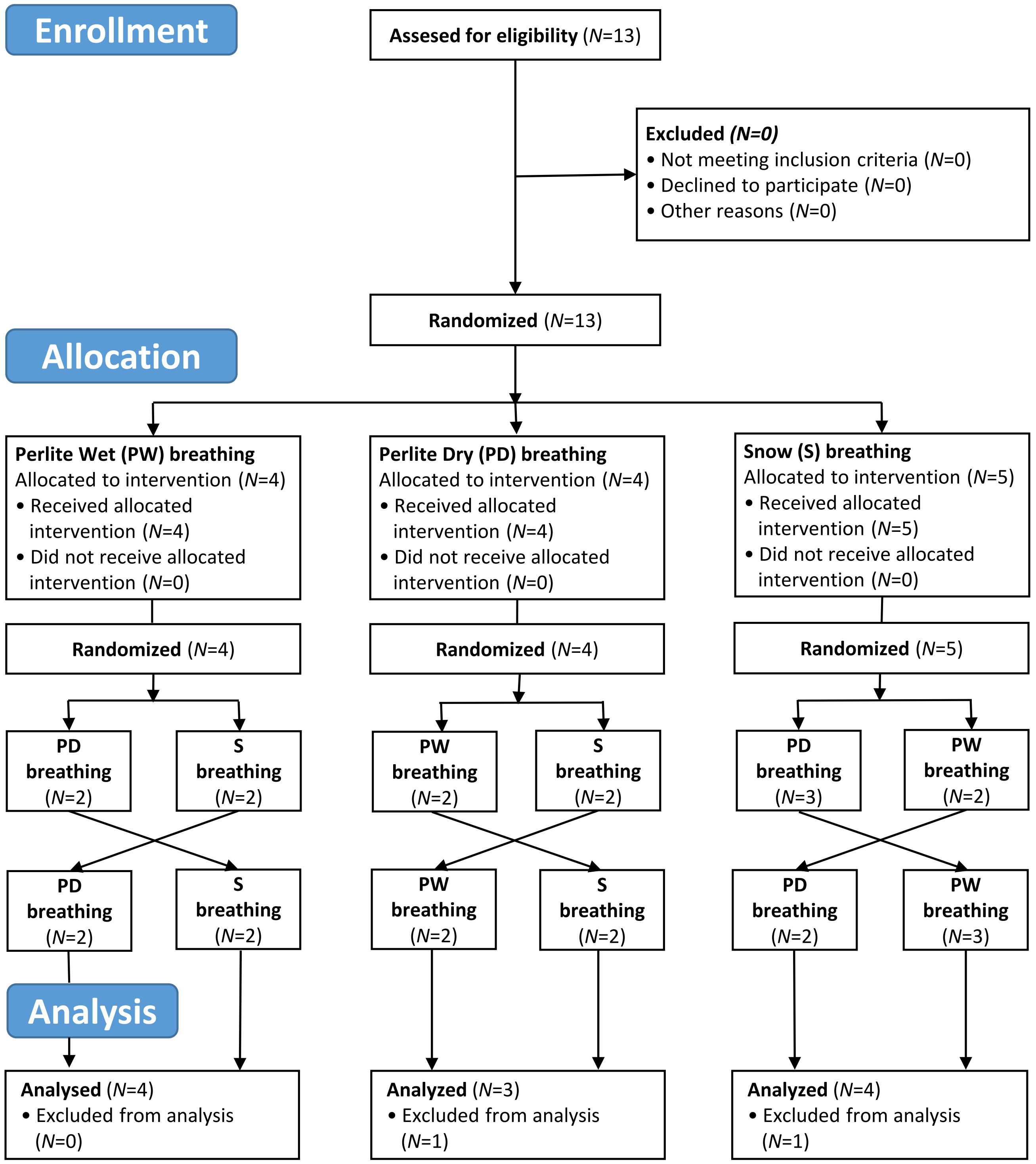

Supplementary figure S1: Flow diagram of the study design with enrollment, allocation and analysis of the participants.
